# MnO_2_ Nanoflower Integrated Optoelectronic Biointerfaces for Photostimulation of Neurons

**DOI:** 10.1002/advs.202301854

**Published:** 2023-06-29

**Authors:** Lokman Kaya, Onuralp Karatum, Rıdvan Balamur, Hümeyra Nur Kaleli, Asım Önal, Sharadrao Anandrao Vanalakar, Murat Hasanreisoğlu, Sedat Nizamoglu

**Affiliations:** ^1^ Department of Electrical and Electronics Engineering Koc University 34450 Istanbul Turkey; ^2^ Research Center for Translational Medicine Koc University 34450 Istanbul Turkey; ^3^ Department of Biomedical Science and Engineering Koc University 34450 Istanbul Turkey; ^4^ Department of Physics Karmaveer Hire College (Shivaji University) 416 209 Gargoti India; ^5^ Department of Ophthalmology School of Medicine Koc University 34450 Istanbul Turkey

**Keywords:** neural stimulation, manganese oxide, photostimulation, supercapacitors

## Abstract

Optoelectronic biointerfaces have gained significant interest for wireless and electrical control of neurons. Three–dimentional (3D) pseudocapacitive nanomaterials with large surface areas and interconnected porous structures have great potential for optoelectronic biointerfaces that can fulfill the requirement of high electrode‐electrolyte capacitance to effectively transduce light into stimulating ionic currents. In this study, the integration of 3D manganese dioxide (MnO_2_) nanoflowers into flexible optoelectronic biointerfaces for safe and efficient photostimulation of neurons is demonstrated. MnO_2_ nanoflowers are grown via chemical bath deposition on the return electrode, which has a MnO_2_ seed layer deposited via cyclic voltammetry. They facilitate a high interfacial capacitance (larger than 10 mF cm^−2^) and photogenerated charge density (over 20 µC cm^−2^) under low light intensity (1 mW mm^−2^). MnO_2_ nanoflowers induce safe capacitive currents with reversible Faradaic reactions and do not cause any toxicity on hippocampal neurons in vitro, making them a promising material for biointerfacing with electrogenic cells. Patch‐clamp electrophysiology is recorded in the whole‐cell configuration of hippocampal neurons, and the optoelectronic biointerfaces trigger repetitive and rapid firing of action potentials in response to light pulse trains. This study points out the potential of electrochemically‐deposited 3D pseudocapacitive nanomaterials as a robust building block for optoelectronic control of neurons.

## Graphical abstract

3D MnO_2_ nanoflowers are successfully integrated into flexible optoelectronic biointerfaces to achieve efficient neuronal stimulation using low‐intensity light. The nanoflowers exhibit high interfacial capacitance at the electrode‐electrolyte interface, enabling the generation of substantial photogenerated charge density for safe and effective photo‐electrical stimulation. These results emphasize the significant potential of 3D pseudocapacitive nanomaterials as a robust platform for optoelectronic control of neurons.

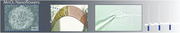

## 1 Introduction

Bioelectronic medicine uses electrical signals to stimulate or silence neurons for the treatment of a wide variety of neurological diseases such as paralysis, Parkinson's disease, and epilepsy.^[^ ^1^ ^]^ Fundamentally electrical currents in the body are generated by the flow of the ions such as sodium (Na^+^) and potassium (K^+^) because of the induced potential differences between the active and return electrodes.^[^ ^2^ ^]^ Although the conventional way to control neural activity is to directly apply electrical potentials by leads, there is an active search for wireless and less invasive electrical stimulation alternatives.^[^ ^3^ ^]^ For that, light provides a noninvasive and spectrally multiplexable trigger option with exceptional temporal and spatial resolution. Moreover, light can be absorbed by photodiodes, create photogenerated charge carriers and induce an electrical potential difference in cellular environments via optoelectronic neural interfaces for the photostimulation of neurons.

Optoelectronic neural interfaces have drawn significant attention for wireless modulation of a wide variety of tissues, such as the heart, brain, and peripheral nerves.^[^ ^3^, ^4^ ^]^ Even in the clinics, they have already started to show vital benefits against blindness caused by macular degeneration, and the photostimulation of secondary neurons in the retina led to the partial recovery of sight.^[^ ^5^ ^]^ For that, rigid photovoltaic devices made of silicon were beneficially used. One of the challenges is to develop flexible optoelectronic neural interfaces that can effectively inject high ionic charge levels via a capacitive charge injection mechanism. Since the bending stiffness is proportional to the cube of the thickness, the use of photoactive materials that have a high absorption coefficient is critical for fabricating flexible devices.^[^ ^6^ ^]^ So far, flexible optoelectronic neural interfaces have been realized via organic polymers, indigo dyes, and quantum dots.^[^ ^4^, ^7^ ^]^ Even though they showed safe capacitive photocurrents, these devices fall short on the injected charge levels because of the fast spikes induced by the double layer capacitance. To increase the charge injection levels, planar PEDOT:PSS, TiN, and RuO_2_ were used.^[^ ^8^ ^]^ Alternatively, 3D pseudocapacitive nanostructures with enhanced electrode‐electrolyte interactions have a high potential for neural interfaces.

In this study, we integrate MnO_2_ nanoflowers (NFs) into a flexible optoelectronic biointerface for the photostimulation of neurons. A MnO_2_ seed layer is initially formed via cyclic voltammetry, and then the nanoflowers are grown on the seed layer via chemical bath deposition. The nanoflower morphology resulted in a high interfacial capacitance that is larger than 10 mF cm^−2^ in a biological media and a high photogenerated charge density over 20 µC cm^−2^ under a low light intensity of 1 mW mm^−2^. Besides the advantageous properties of MnO_2,_ such as high capacitance per unit mass,^[^ ^9^ ^]^ wide operational potentials,^[^ ^10^ ^]^ and being an abundant raw material,^[^ ^11^ ^]^ we observed that MnO_2_ nanoflowers do not induce any toxicity on the hippocampal neurons. We did patch‐clamp electrophysiology of hippocampal neurons on the biointerface under whole‐cell recording, and we observed the repetitive firing of neurons under the illumination of pulse trains. Our study highlights the 3D nanomorphology of pseudocapacitive materials as a promising tool for the optoelectronic control of neurons.

## 2 Results and Discussion

### 2.1 Device Fabrication

**Figure 1:**
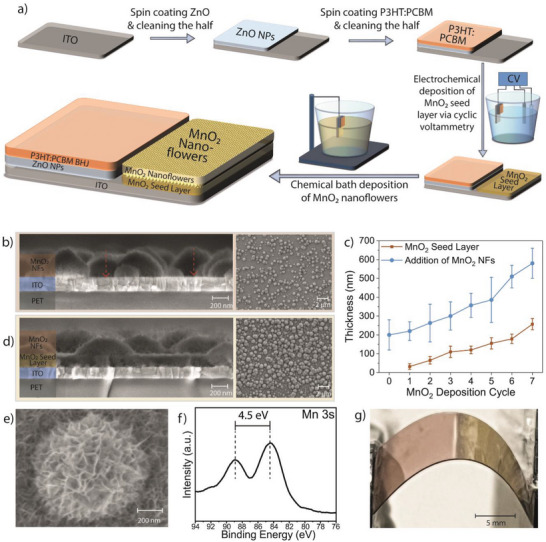
a) The fabrication flow schematic of MnO_2_ nanoflower integrated optoelectronic biointerface. (Not drawn in scale). b) (Left) Cross‐sectional FESEM (field emission scanning electron microscopy) of the return electrode without MnO_2_ seed layer. The red arrows show the regions that create unwanted electrical pathways between the ITO layer and the electrolyte. (Right) Top FESEM view of the structure. c) The thickness of the MnO_2_ seed layer and nanoflower‐modified seed layer as a function of the seed layer deposition cycle. d) (Left) Cross‐sectional FESEM of the return electrode after the nanoflower growth on the seed layer. (Right) Top FESEM view of the structure. e) Top FESEM view of a MnO_2_ nanoflower. f) High resolution XPS spectrum of Mn 3s. g) Photograph of the flexible optoelectronic biointerface. The left half (pinkish) is the photoactive part, and the right half (yellowish) is the return electrode with nanoflowers.

The optoelectronic biointerfaces are fabricated by sequential spin coating and cleaning cycles for the photoactive part, which is followed by nanoflower synthesis on the return electrode (**Figure** 1a). First, ZnO nanoparticles (NPs) are spin‐coated on indium tin oxide (ITO), and annealing is done to form a uniform NP layer. Then, poly(3‐hexylthiophene‐2,5‐diyl):[6,6]‐phenyl‐C61‐butyric acid methyl ester (P3HT:PCBM) bulk heterojunction (BHJ) thin film is spin‐coated (Figure S1, Supporting Information).^[^ ^12^ ^]^ After each step, half of the device is cleaned to keep the return electrode area uncoated. Thus, the photoactive part of the neural interface is formed on one half of the device, while unmodified ITO is on the other half. Afterward, MnO_2_ nanoflowers are directly formed via chemical bath deposition on top of the ITO (Figure 1b). However, the direct formation of MnO_2_ NFs leads to incomplete coverage of the ITO layer, which forms unwanted shunt impedance at the electrode‐electrolyte interface and decreases the interfacial capacitance (Figure S2, Supporting Information).

As a solution, the MnO_2_ seed layer is initially formed on ITO via cyclic voltammetry (CV) deposition, and afterward, the nanoflowers are grown via chemical bath deposition. The seed layer is deposited by submerging the return electrode region (i.e., ITO) into MnSO_4_: Na_2_SO_4_ aqueous solution.^[^ ^13^ ^]^ The thickness of the seed layer can be adjusted by increasing the CV cycle (Figure 1c). After each cycle, an average thickness of 33 nm (±12 nm) is deposited, and ITO is homogeneously covered after five cycles of seed layer deposition with a mean thickness of 110 nm (±30 nm). Then, to form nanoflowers on the seed layer, a MnO_2_ nanoflower layer is formed on top of the seed layer by chemical bath deposition via immersing the return electrode into the highly concentrated Na_2_SO_4_: MnSO_4_:K_2_S_2_O_8_ aqueous solution.^[^ ^14^ ^]^ Once this step is completed, MnO_2_ nanoflowers (with a mean size distribution of 279 ± 169 nm) (Figure S3, Supporting Information) are formed onto the MnO_2_ seed layer. The MnO_2_ seed layer facilitates enhanced nanoflower density during chemical bath deposition while the chemical bath deposition parameters, as in the case of nanoflower growth on ITO, were kept constant (Figure 1d). Advantageously, the FESEM image of a single MnO_2_ NF on the seed layer illustrates the nanomorphology with a high surface area (Figure 1e). Moreover, the MnO_2_ seed layer facilitated a complete MnO_2_ and electrolyte interface without ITO. X‐ray photoelectron spectroscopy (XPS) was performed to study the composition of the manganese oxide nanoflowers (Figure S4, Supporting Information). To better understand the oxidation states of the manganese, we also conducted the XPS analysis in the Mn 3s region (Figure 1f). The Mn 3s spectrum exhibited two peaks located at 84.4 and 88.9 eV. The multiplet splitting of the Mn 3s peaks was 4.5 eV, which corresponds to mainly Mn (IV) oxidation states indicating MnO_2._^[^ ^15^ ^]^ The whole solution‐processed fabrication procedure can be directly used to fabricate devices on flexible substrates (e.g., PET) (Figure 1g), which is beneficial to have conformal contact with tissues.^[^ ^4^, ^7^ ^]^

### 2.2 Electrochemical Characterization

**Figure 2:**
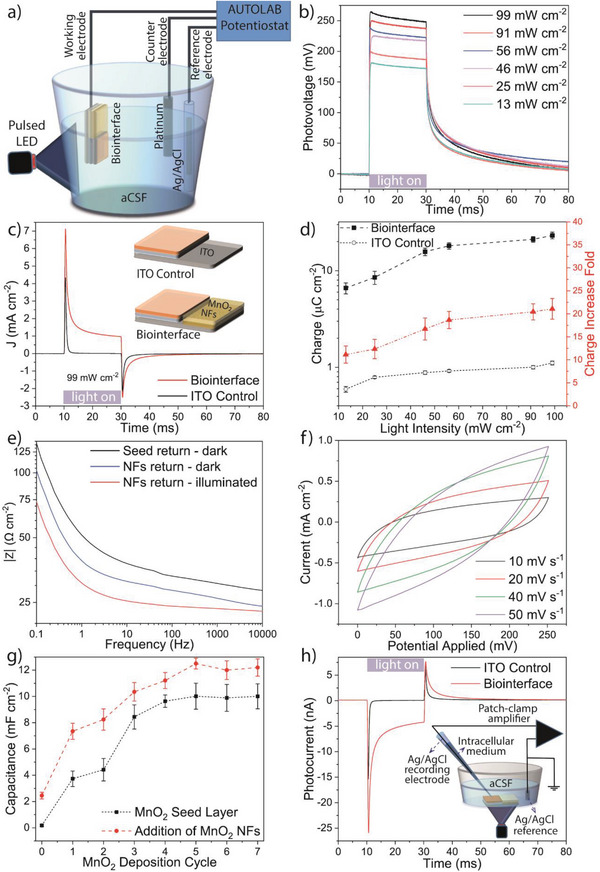
a) The schematic of the three‐electrode AUTOLAB potentiostat for photocurrent and photovoltage characterization of the optoelectronic biointerfaces. b) Photovoltage of the biointerface under different light intensity levels (*λ* = 450 nm). c) Photocurrent density of the biointerface and ITO control (i.e., biointerface with ITO return electrode) under the illumination of 99 mW cm^−2^. Inset shows the schematic of the measured devices. d) Charge injection density of the biointerface and ITO control in aCSF (left axis) and fold of charge increase of the biointerface compared to ITO control (right axis) under different light intensities (mean ± SD for *n* = 10). e) Bode modulus of the seed layer and return electrode with nanoflowers under dark and light (99 mW cm^−2^) in the frequency range of 0.1 Hz–10 kHz. f) Cyclic voltammograms of the return electrode in the range of 0–250 mV as a function of scan rate. g) The capacitances of the return electrodes on the seed layer with and without nanoflowers. h) The measured photocurrents of the biointerface and ITO control over the surface of the photoactive regions under the illumination of 99 mW cm^−2^. Patch‐clamp pipettes in the electrophysiology setup were used without any wire connection. Inset shows the simplified schematic of the measurement configuration.

To quantify the contribution of MnO_2_ NFs on the overall device performance, we measured the short circuit photoresponse via a potentiostat/galvanostat instrument. For that, we utilize an electrochemical setup with three electrodes where the working electrode is connected to the ITO, the Ag/AgCl is used as the reference electrode, and the platinum is the counter electrode (**Figure** **2**a). The photovoltage of the optoelectronic biointerface and the control group (without MnO_2_) are quantified under 20 ms light pulses with various light intensity levels (Figure 2b). In our electrochemical system, we used the biological fluid of artificial cerebrospinal fluid (aCSF) solution to understand the working performance in physiological conditions. We observed the photovoltages of 260 mV under the light illumination with the intensity of 99 mW cm^−2^, which is comparable to the previously reported retina implants.^[^ ^8a^ ^]^

In Figure 2c, the current density per area of the biointerface is shown, and in comparison with the control group, the MnO_2_ NF integration facilitates a 21‐fold increase of the ionic charge levels, which is a significant improvement for the effective photostimulation of neurons. Though the photocurrent peak was increased only 1.5‐fold, the increment in the charge was drastically enhanced by more than 20‐fold because of the slower decay of the photocurrent. We calculated the responsivity (peak photocurrent/illuminated light power) and the external quantum efficiency (number of generated ionic charges/externally applied number of photons) of the device. The responsivity corresponds to 70.53 mA W^−1^, and the external quantum efficiency for a 20 ms light pulse is 0.034 ion per photon. We investigated the injected charge density for the biointerface and the control under various light intensities (Figure 2d), and the biointerface generates significantly higher charge densities compared to the control. The charge density levels are sufficient to stimulate a wide variety of human tissue in vivo, such as the human subthalamic nucleus, sub‐retina, and cortical part of the brain.^[^ ^16^ ^]^ Figure 2e shows that the impedance is decreased after the addition of NFs under dark. In comparison with the seed layer, the decrease of the impedance originates from the branched walls of nanoflowers with a larger effective surface area because of the nanosized petals and conduits. Moreover, the impedance further decreases under illumination.

In order to evaluate the charge generation and storage behavior, the cyclic voltammograms (CV) of NFs within the device operation potentials were recorded, and they reveal capacitive‐like current–voltage behavior (Figure 2f). The quasi‐rectangular shapes of the CV show the presence of reversible redox reactions and the pseudocapacitive behavior of the interface, which is critical for the safe photostimulation of neurons and for keeping the cellular environment healthy for cells. Here the reversibility of the Faradaic reactions originates from the surface adsorption and intercalation/deintercalation of electrolyte cations of Na^+^ and K^+^ that are involved in the aCSF solution.^[^ ^17^ ^]^ Moreover, since the operation range of our device is within the working potential window (WPW) of MnO_2_ (−0.2–1.2 V), this suppresses irreversible hydrogen and oxygen redox reactions.^[^ ^10^ ^]^ We checked the photoactivity of the MnO_2_ NFs, and observed that it could not induce any photocurrent (Figure S5, Supporting Information). Thus, the increase in the photocurrent and charge injection density is solely due to the pseudocapacitive behavior of the nanostructured return electrode.

To quantify the capacitance levels, we integrated the area under the CV curves (Figure S6, Supporting Information). The seed layer has a direct effect on the capacitance, which increases with the number of deposition cycles and saturates due to the limited diffusion of the electrons after five cycles. On top of the seed layer, the growth of MnO_2_ NFs increases the capacitance of the return electrode structure because of the enhanced surface area (Figure 2g and Figure S6, Supporting Information). During its operation in a cellular environment, the biointerface stimulates neurons without any wire connection; hence the waveshapes and the increase of the charge injection levels are also confirmed by electrophysiology measurement setup under such a wireless condition, which consists of a recording electrode inside a thin glass pipette filled with intracellular solution and a distant reference bath electrode in the aCSF with a patch clamp amplifier (Figure 2h inset).^[^ ^18^ ^]^ Likewise, the peak of photocurrent was also increased 1.5‐fold, and a similar waveshape of the device was observed (Figure 2h).

### 2.3 Stability and Biocompatibility

**Figure 3:**
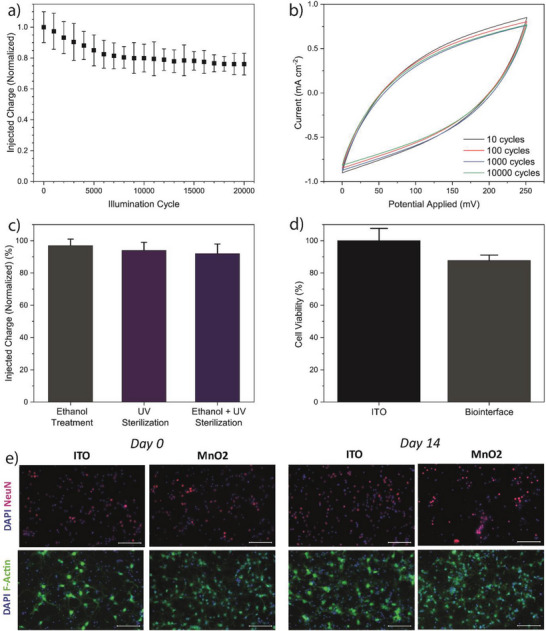
a) Charge injection stability of MnO_2_ optoelectronic biointerface for 20 000 photo‐cycles (mean ± SD for *n* = 5). Illumination parameters are 450 nm wavelength, 20 ms pulse width, 1 Hz illumination frequency, and 99 mW cm^−2^ light intensity. b) Cyclic voltammogram stability of nanoflowers up to 10 000 cycles. Measurement parameters are 40 mV s^−1^ scan rate and 0–250 mV potential window. c) Change in normalized charge injection after sterilization steps of the optoelectronic biointerface (mean ± SD for *n* = 4). d) CTG cytotoxicity assay for quantifying cell viability of primary hippocampal neurons cultured on the biointerface and control ITO devices (mean ± SEM for *n* = 4). An unpaired, two‐tailed *t*‐test was used for statistical analysis, and *p < 0.05 was evaluated as statistically significant. e) Immunofluorescence images of cells cultured on the biointerface and control ITO devices on days 0 and 14 to observe the morphology and viability of primary hippocampal neurons. Cells were co‐stained with DAPI (blue) to show the nucleus, Anti‐NeuN (red) to show the neuronal nucleus, and Anti‐f‐Actin (green) to indicate cell structure (scale bar: 100 µm).

We investigate the stability and biocompatibility of the MnO_2_ nanoflower integrated optoelectronic biointerface. The photocyclic stability study reveals that the biointerface has a charge injection performance of 76±7% after 20 000 illumination cycles (**Figure** **3**a). The electrochemical stability of MnO_2_ NFs is evaluated by repetitive CV characterization, and the area enclosed by CV graphs, which is correlated with capacitance, remains the same for the 10 000 cycles showing the good stability of nanoflowers (Figure 3b). One of the critical steps of cellular experiments is the sterilization of the biointerface. We confirm that the biointerface retains 92±6% of its charge injection after the sterilization steps of ethanol and UV treatment (Figure 3c). After stability characterizations, we conduct biocompatibility experiments. The cell viability experiments are performed with primary hippocampal neurons cultured on the biointerface and ITO control substrates. After 3 days of neuron culture on substrates, a CTG assay is performed to examine the cytotoxicity, and the biointerface has comparable viability with the ITO substrates (Figure 3d). Moreover, day 0 and day 14 cultures show short‐ and long‐term morphological changes and neural network improvements on both the biointerface and ITO control substrates. We confirm neuron survival and maintenance of their characteristics on the biointerface after 2 weeks of culture by immunofluorescence staining of anti‐NeuN, a neuronal nuclear protein, and f‐Actin, cytoskeleton filaments (Figure 3e).

### 2.4 Electrophysiology

**Figure 4:**
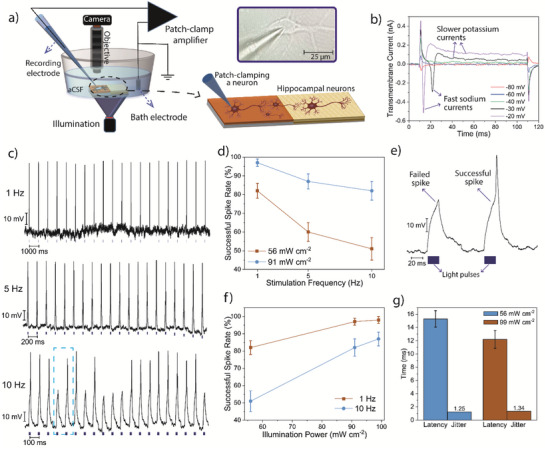
a) The schematic of the patch‐clamp electrophysiology recording setup. Inset (Down): The schematic of patching of a neuron. Inset (Top): Photograph of a patched neuron. b) Transmembrane current of a patched neuron under whole‐cell recording for different holding potential levels. c) Current‐clamp recording of primary hippocampal neurons under photostimulation of different frequencies for 1, 5, and 10 Hz. The wavelength of light is 450 nm, the illumination intensity is 91 mW cm^−2^, and the duration of each pulse is 20 ms (the small rectangles represent light pulses). The area enclosed by dashed blue lines is zoomed in figure 4e. d) Stimulation frequency dependence of successful spike rate for 56 and 91 mW cm^−2^ with a duration of 20 ms (mean ± SD for *n* = 6). e) Failed and successful action potential traces at 10 Hz. f) Illumination power dependence of successful spike rate for 1 and 10 Hz (mean ± SD for *n* = 6). g) Latency and jitter of the biointerface under the illumination of 56 and 91 mW cm^−2^ (pulse duration of 20 ms; frequency of 1 Hz) (mean ± SD for *n* = 6).

To test the light‐induced stimulation of neurons, we execute single‐cell patch clamp recordings with primary hippocampal neurons that are cultured on the biointerface.^[^ ^19^ ^]^ We use an Ag/AgCl electrode in the pipette filled with the intracellular solution as the recording electrode and a distant Ag/AgCl reference electrode (**Figure** **4**a). We record the electrical activity of neurons in the whole‐cell configuration (Figure 4a inset), and the biointerface operates in open‐circuit conditions without any wire connection. First, we check the required membrane depolarization for the stimulation of neurons. For that, the cell membrane potential is held at different holding potentials, and the transmembrane current is recorded in voltage‐clamp mode under dark conditions. The values higher than −40 mV cause the opening of the voltage‐gated sodium channels, which indicates the possibility of the firing of an action potential (Figure 4b). For −20 and −30 mV holding potentials, the fast sodium inward currents are followed by slower potassium outward currents. Moreover, the delay of the sodium gate activation is less for higher potentials due to a larger amount of depolarization in the same time interval.

For the photostimulation of neurons, we apply light‐pulse trains at different frequency levels (1–10 Hz) while recording the membrane potential under current‐clamp mode. At 1 Hz, we can observe highly repetitive and successful action potential generation (Figure 4c (top)). After an initial capacitive spike induced by the light onset, the biointerface continuously injects charges during “light‐on” periods owing to the MnO_2_‐induced reversible electrochemical reactions at the device‐electrolyte interface, which is followed by the second capacitive spike at the light offset (Figure S7, Supporting Information). The charge injection during “light‐on” periods leads to hyperpolarization of the attached membrane and depolarization of the free membrane, which leads to the firing of neurons above the threshold.^[^ ^20^ ^]^ The success rate decreases for identical illumination power as we increase the frequency (Figure 4d,e). To compensate for the decrease in the successful spike rate, we applied higher light intensities, which led to higher success for the photostimulation of neurons (Figure 4d,f). AP latency (time between the start of the illumination and AP peak) and jitter parameters are shown in Figure 4g for different illumination powers. The lower latency for greater light intensity is due to the higher charge density levels in the identical time interval. The latencies are significantly lower compared to other recent forms of optical stimulation techniques, such as photothermal and photoacoustic stimulation,^[^ ^21^ ^]^ and similar to the range observed in optogenetics.^[^ ^22^ ^]^ Since certain neural activities have millisecond or sub‐millisecond spike timing precisions,^[^ ^23^ ^]^ such latency and jitter levels are advantageous for inducing temporally precise action potentials and minimizing the temporal variabilities across neurons in order to examine neural mechanisms with higher accuracy.^[^ ^24^ ^]^

### 2.5 Discussion

This study demonstrated the successful integration of nanoflowers into flexible optoelectronic biointerfaces for effective photostimulation of neurons. In addition to nanoflowers, other nanomorphologies such as nanocubes, nanowires, nanorods, and hollow spheres can also be deposited using a similar electrochemical approach.^[^ ^17^ ^]^ Furthermore, MnO_2_ can be chemically modified by mixing it with other transition metal elements and doping it with metallic elements such as Al, Sn, and Pb, providing tunability of electrical properties for cell interfaces.^[^ ^25^ ^]^ Tuning nanomorphology and electrical properties holds high potential for developing pixilated device structures with microscale stimulation electrodes. As the electrode size decreases, the impedance increases, and the charge injection capacity decreases. To overcome this issue, the use of materials with high reduction‐oxygen capacity and electrochemical surface area is necessary.^[^ ^26^ ^]^ Therefore, the integration of pseudocapacitive materials with enhanced surface area via 3D nanostructures has a high potential for developing miniaturized and flexible bioelectronic devices.

MnO_2_ nanoflowers beneficially show a lower impedance in comparison with our previous studies using RuO_2_.^[^ ^8b^ ^]^ MnO_2_ nanoflowers also decrease the impedance in comparison with its seed layer as well (Figure S8, Supporting Information). In comparison with the previous state of the art organic optoelectronic biointerfaces, the optimized device can facilitate a higher photocurrent and charge level (Table S1, Supporting Information). In addition, photoresponse under near‐infrared (NIR) light illumination within the tissue transmission window can be obtained by replacing the photoactive layer with NIR‐responsive organic or inorganic materials such as PCPDTBT:PC60BM^[^ ^27^ ^]^ or PbS quantum dots,^[^ ^7c^ ^]^ respectively.

Alternative to ITO, MnO_2_ nanoflowers can be also formed on FTO, gold, and stainless steel (Table S2, Supporting Information). To demonstrate this, we conducted an experiment on FTO substrates and obtained comparable photocurrent, photovoltage values, and similar capacitance values for the MnO_2_ return electrode. For MnO_2_ coating on FTO, we used 12 mV s^−1^ and six deposition cycles, which is followed by the chemical bath deposition. Additionally, we deposited the MnO_2_ seed layer on gold and stainless steel substrates with a slight alteration in electrochemical deposition parameters. Since the thin gold layer is more conductive, we decrease the step voltage to 4 mV s^−1^ and the deposition cycle to 2 with the same deposition duration. Similarly, for stainless steel mesh, we used 2 mV s^−1^ step voltage and one deposition cycle. For all the substrates, the chemical bath deposition duration remained the same with a slight alteration in bath temperature and annealing temperature (Table S2, Supporting Information). The formation of MnO_2_ nanoflower is also visualized by SEM (Figure S9, Supporting Information). Other than these substrates, MnO_2_ flowers can also be obtained on graphene, graphite, and zeolitic imidazolate framework.^[^ ^28^ ^]^

There are other materials that can be used in nanoflower form, such as nickel hydroxide (Ni(OH)_2_),^[^ ^29^ ^]^ cobalt oxide (Co_3_O_4_),^[^ ^30^ ^]^ copper oxide (CuO),^[^ ^31^ ^]^ zinc oxide (ZnO),^[^ ^32^ ^]^ and ruthenium oxide (RuO_2_).^[^ ^33^ ^]^ Even though Ni(OH)_2_ and Co_3_O_4_ have higher capacitance, however, they are not suitable for in vivo applications as Ni(OH)_2_ is highly toxic, and Co_3_O_4_ can release significant amounts of ROS (reactive oxygen species) during in vivo applications.^[^ ^34^ ^]^ The remaining metal oxides are the best contenders as supercapacitor electrode materials for bioelectronics owing to their wide variety of oxidation states, which are suitable for safe charge transfer. Compared to CuO and ZnO, MnO_2_ and RuO_2_ have higher capacitance but the high impedance of metal oxides generally presents a drawback.^[^ ^35^ ^]^ To decrease the impedance a nanoflower morphology can be added (Figure 2e). Since the formation of RuO_2_ nanoflowers is comparatively much longer and complex,^[^ ^33^ ^]^ we selected MnO_2_ material for nanoflowers that can be deposited to the return electrode in a few hours with a simple fabrication procedure. In addition, MnO_2_ is an ideal electrode material due to its non‐toxic nature, abundance, low cost, wide potential range, high electrochemical activity, high theoretical capacity (1370 F g^−1^), adjustable thickness, and shape via coating method and parameters. Its biocompatibility is another advantage for biointerface applications. Therefore, we selected MnO_2_ for neural stimulation.

MnO_2_ is a well‐known material for energy storage devices,^[^ ^36^ ^]^ and its nanostructures have recently attracted significant attention for biomedical applications. For instance, MnO_2_‐based nanoscaffolds have been found to enhance the adhesion of stem cells, promote their differentiation into neurons, and facilitate neurite outgrowth for stem cell transplantation.^[^ ^37^ ^]^ Furthermore, MnO_2_ nanoparticles possess immunomodulatory properties for cancer starvation therapy and can induce oxidative stress and autophagy in nutrient‐deprived cancer cells.^[^ ^38^ ^]^ Additionally, MnO_2_ nanosheets have been utilized for biocatalysis, fluorescence quenching in biosensing, and MRI‐contrast agents.^[^ ^39^ ^]^ Therefore, this first study, employing MnO_2_ nanostructures for the photostimulation of neurons, can be combined with different forms of MnO_2_ for multifunctional stimulation, sensing, and therapy.

In summary, the integration of 3D pseudocapacitive nanomaterials into optoelectronic biointerfaces shows great promise for wireless and electrical control of neurons. This study shows the successful integration of manganese dioxide nanoflowers into a flexible optoelectronic biointerface that can safely and efficiently stimulate neurons with low light intensity. The nanoflowers facilitated a high interfacial capacitance and photogenerated charge density, inducing reversible Faradaic reactions that did not cause any toxicity to hippocampal neurons in vitro. These findings suggest that 3D pseudocapacitive nanomaterials, such as MnO_2_ nanoflowers, could serve as a reliable building block for future optoelectronic control of neurons. Furthermore, the ability of the optoelectronic biointerfaces to trigger repetitive and rapid firing of action potentials in response to light pulse trains can have significant applications in the fields of neuroprosthetics.

## 3 Experimental Section

### Device Fabrication

Photoelectrodes were fabricated on glass or PET substrates covered with indium tin oxide (ITO). The glass/ITO (Ossilla, S111) or PET/ITO (Sigma Aldrich, 639 303) substrates were cleaned in an ultrasonic cleaner system at 50 °C with immersing consecutively in a sodium hydroxide (NaOH) solution for 5 min, tension‐active agent (HELLMANEX III):deionized water (3%, 97% by volume) for 15 min, deionized water for 15 min (repeated two times to remove access HELLMANEX), acetone for 10 min, and isopropanol for 10 min. After oven drying at 60 °C for 30 min, the substrates were treated with UV ozone for 20 min to eliminate any other residues on the ITO surface. The ZnO precursor solution was prepared by mixing 110 mg of zinc acetate dehydrate (Zn‐(CH_3_CO_2_)_2_·2H_2_O) (Sigma‐Aldrich) in 1 mL of 2‐methoxyethanol (C_3_H_8_O_2_), and 42 mg of ethanolamine (HOCH_2_CH_2_NH_2_) and the mixture is stirred for 5 min. Then the ZnO solution was sonicated for 2 h at 50 °C and filtered through a 0.45 µm PVDF filter. The P3HT (95.7% regioregular, >99% pure, Ossilla) and PCBM (>99% pure, Ossilla) solutions were prepared by mixing the donor material (P3HT) and the acceptor material (PCBM) solutions with the blending ratio of 1:1. P3HT and PCBM solutions were prepared separately in o‐dichlorobenzene with optimized concentrations of 60 mg mL^−1^ (Figure S1, Supporting Information), stirred at 50 °C for 8 h, and then mixed and stirred at 50 °C for 3 h using a magnetic stirrer. The ZnO solution was spin‐coated on ITO substrates at 2000 rpm for 60 s, and half of the substrate was cleaned by wiping with cotton swabs soaked with methanol. Then, the substrates were annealed at 280 °C for 15 min for glass/ITO substrates and 200 °C for 30 min for PET/ITO substrates. Later, The photoactive layer was fabricated by spin‐coating the P3HT:PCBM blend onto the ZnO layer at 1000 rpm for 60 s, and half of the substrate was cleaned by wiping with cotton swabs soaked with toluene. Later, the devices were annealed at 150 °C for 10 min to form P3HT:PCBM bulk heterojunction. After these steps, the photoactive part of the neural interface is formed on one half of the device, while unmodified ITO is on the other half. To form the MnO_2_ return anode electrode on the intact ITO region, first, the MnO_2_ seed layer was electrodeposited by submerging the ITO return electrode region into MnSO_4_:Na_2_SO_4_ (5 mm:0.1 m) aqueous solution at room temperature and performing CV measurement for 5 cycles with 10 mV s^−1^ sweep rate from 0.4 to 1.3 V with respect to Ag/AgCl bath electrode.^[^ ^13^ ^]^ The devices were rinsed in distilled water and annealed at 50 °C for 30 min. For gold substrates in this step, we needed to decrease the step rate to 4 mV s^−1^ and deposition cycle to 2 to get as gold is more conductive, and a lower step rate ensures the current is not overloaded and stays within a few mA range during CV coating. Similarly, for stainless steel mesh, we used 2 mV s^−1^ step voltage and 1 deposition cycle due to high conductivity and high surface area of the substrate. For FTO substrates, due to higher impedance, we increased step voltage to 12 mV s^−1^ and deposition cycle to 6, while the annealing temperature was increased to 80 °C degree to ensure better attachment of the seed layer due to the higher thickness of FTO substrates. Then, to form a high‐surface‐area porous oxide layer for obtaining a large return electrode capacitance, the MnO_2_ NFs layer was formed on top of the MnO_2_ seed layer by chemical bath deposition. In this technique, the MnO_2_ seed layer was immersed into the highly concentrated Na_2_SO_4_:MnSO_4_:K_2_S_2_O_8_ (0.33 m:0.33 m:0.33 m) aqueous solution ^[^ ^14^ ^]^ for 2 h at 60 °C to form nanoflowers on the return electrode region, then they are annealed at 70 °C for ITO substrates. Chemical bath deposition duration remained the same for all substrates with a slight alteration in bath temperature and annealing temperature to have similar morphologies and ensure attachment of nanoflowers (Table S2, Supporting Information). These parameters can be adjusted for the targeted morphology and nanoflower shapes.^[^ ^14^ ^]^ The MnO_2_ nanoflowers fabricated on FTO, gold, and stainless steel substrates show similar morphologies compared to ITO substrates (Figure S9, Supporting Information). Other than these substrates, MnO_2_ flowers can be also obtained on graphene, graphite, and zeolitic imidazolate framework.^[^ ^28^ ^]^

### X‐Ray Photoelectron Spectroscopy Analyses

XPS analyses of MnO_2_ nanoflowers were carried out by a Thermo Scientific K‐Alpha XPS with Al K‐alpha monochromatic radiation (1486.3 eV). For XPS measurements, dried samples were exposed to 400 µm X‐ray spot size and 50.0 eV pass energy. The experimental pressure and the base pressure were kept below about 1 × 10^−7^ and 3 × 10^−9^ mbar, respectively. The C 1s peak at 285.0 eV was designated for the assessment.

### Photoelectrochemical Measurements

An Autolab Potentiostat Galvanostat PGSTAT system (Metrhom, Netherlands) was used for photoelectrochemical measurements, such as chronoamperometry, chronopotentiometry, cyclic voltammetry, and electrochemical impedance characterization. The three‐electrode system was used with a platinum rod as the counter electrode, Ag/AgCl as the reference electrode, and a connection to the ITO layer of the BI as the working electrode. 1 cm^2^ of the biointerface with the other two electrodes was immersed in an extracellular aCSF medium that mimics in vivo environment as an electrolyte solution at room temperature. The aCSF medium was prepared by mixing 10 mm of 4‐(2‐hydroxyethyl)‐1‐piperazineethanesulfonic acid (HEPES), 10 mm glucose, 2 mm CaCl_2_, 140 mm of NaCl, 1 mm of MgCl_2_, and 3 mm of KCl. The pH of aCSF solution was adjusted to 7.4 by adding the required amount of NaOH solution. Then, a blue LED (M450LP1, Thorlabs) was used as the light source. The LED was driven by an LED Driver with Pulse Modulation (DC2200‐High‐Power 1‐Channel, Thorlabs) to produce a train of 20 ms light pulses. Then, the data was recorded and analyzed using the NOVA software. An optical power meter (Newport 843‐R) was utilized to measure the optical power densities of incident light on the biointerface.

### Primary Neuron Isolation

All experimental procedures were approved by the Institutional Animal Care and Use Committees of Koç University (Approval No: 2021.HADYEK.022) according to Directive 2010/63/EU of the European Parliament and of the Council on the Protection of Animals Used for Scientific Purposes. For isolation of primary hippocampal neurons, the brains of Wistar Albino rats at embryonic day 15–17 (E15‐E17) were dissected, and brains were cleared of blood vessels and the meninges. Then hippocampi were obtained and placed in ice‐cold Hank's Balanced Salt Solution (HBSS, Thermo Fisher Scientific, MA, USA) supplemented with glucose and sucrose. The hippocampi were incubated in 0.25% Trypsin‐EDTA solution (Thermo Fisher Scientific, MA, USA) supplemented with 2% DNase‐I (NeoFroxx, Einhausen, Germany) for 20 minutes in a 37 °C incubator. After incubation, Dulbecco's Modified Eagle Medium/Nutrient Mixture F‐12 (DMEM/F12 Thermo Fisher Scientific, MA, USA), including 10% fetal bovine serum (FBS, Heat Inactivated, GE Healthcare, IL, USA) and 1% penicillin/streptomycin (Thermo Fisher Scientific, MA, USA) was added to inhibit excess enzyme activity and tissue suspension was centrifuged. The supernatant was removed, and freshly prepared plating Neurobasal Medium (NBM, Thermo Fisher Scientific, MA, USA) including 2% B27, 1% L‐glutamine, 1% penicillin/streptomycin, *β*‐mercaptoethanol, glutamate was added to the pellet. The remaining tissue was mechanically dissociated by trituration with a Pasteur pipet, and cells were collected in a falcon tube after passing through a 70 µm cell strainer. The homogenous cell solution was seeded on poly‐l‐lysine (PLL, Sigma‐Aldrich, MO, USA) coated ITO and MnO_2_ substrates. Cells were incubated for 3 days at 37 °C incubator with 5% CO_2_; then, the media was changed with NBM supplemented with cytosine arabinoside (Sigma‐Aldrich, MO, USA) to eliminate glia proliferation. After glia removal, fresh NBM was replaced to maintain culturing until experiments.

### Electrophysiology Experiments

The recording of the electrical activity of cultured hippocampal neurons was conducted by utilizing EPC 800 Heka Elektronik patch‐clamp amplifier system (Pfalz, Germany). In the whole‐cell configuration, the voltage‐clamp and current‐clamp recordings were performed for single‐cell transmembrane current and transmembrane voltage recordings, respectively. The recorded cells were on the photoactive region of the BI that was submerged in aCSF extracellular medium without any wire connection. The reference Ag/AgCl bath electrode was far from the BI to mimic in vivo conditions. The patch‐pipettes with 6–8 MΩ resistance were prepared by filling them with intracellular medium, which consisted of 140 mm KCl, 2 mm MgCl2, 10 mm HEPES, 10 mm ethylene glycol‐bis (*β*‐aminoethyl ether)‐*N,N,N′,N′‐*tetraacetic acid (EGTA), 2 mm Mg‐ATP, and a required amount of KOH to adjust the pH to 7.2, in distilled water. The current‐clamp data was subjected to downsampling to reduce the computational complexity for statistical analysis of action potentials while preserving the integrity of the data (Figure S7, Supporting Information). The goal was to conduct a meaningful analysis of the action potentials without sacrificing any vital information. A digital camera integrated with an Olympus T2 upright microscope was used to observe the patch pipette and cells. Photocurrent analysis was conducted using the identical system with the patch‐clamp amplifier in voltage‐clamp mode, and the biointerface was not cultivated with cells yet. The pipette was 100 micrometers above the surface of the biointerface and had 4–6 MΩ resistance for the measurement.

### Biocompatibility Assay

CellTiter‐Glo Luminescent‐based cell viability assay (CTG, Promega, Mannheim, Germany) was used to quantify alive cells on the control and MnO_2_ biointerfaces. This technique provides quick results to detect the number of viable cells depending on the amount of ATP in cell populations. Isolated primary hippocampal neurons were seeded as 500 000 cells well^−1^ on PLL‐coated devices and cultured as explained above. To determine cell viability, a 1:1 ratio of CTG solution was added to cells and shaken for 2 min on an orbital shaker. Then cells were allowed for 10 min of incubation at RT to stabilize luminescence signals. After incubation, the solution was transferred to a 96‐well opaque plate, and the luminescence signals(RLU) were measured with Synergy H1 Microplate Reader (Bio‐Tek Instruments). The relative cell viability of each sample was calculated as follows: Viability = (RLUsample/RLUcontrol) × 100. The luminescence of the sample was obtained from the cells grown on the MnO_2_ device, and the luminescence of the control was obtained from the cells grown on the ITO control substrates.

### Immunofluorescence Staining and Imaging

Primary hippocampal neurons were seeded as explained above on the ITO control, and the MnO_2_ substrates as 500 000 cells substrate^−1^. The short‐term effect of substrates on cells was checked at Day 0, and long‐term alteration on neuron characteristics and morphology were monitored on the 14th day of culture. The neurons at Day 0 or Day 14 were fixed by ice cold 4% paraformaldehyde in PBS for 20 min and washed three times with PBS‐T (Phosphate Buffered Saline, 0.1% Tween‐20). For permeabilization, the cells were incubated with 0.1% TritonX‐100 in PBS for 8 min. Then, they were incubated with Superblock (Thermo Fisher Scientific, MA, USA) for 10 min at room temperature. For neuron characterization, primary hippocampal neurons were incubated overnight with an anti‐NeuN antibody (ab177487, Abcam, Cambridge, UK) prepared in a blocking solution. After incubation, samples were washed with PBS‐T, and they were incubated with goat anti‐rabbit IgG H&L Alexa Fluor 488 (Cell Signaling Technology, MA, USA) to label anti‐NeuN for 90 min at 37 °C. To show the cytoskeleton of neurons grown on either the control substrate or MnO_2_ device, cells were stained with FITC‐conjugated phalloidin antibody for 90 min at 37 °C. All samples were washed three times with PBS‐T, then mounted with DAPI supplemented mounting medium (50 001, Ibidi GmbH, Germany). All immunofluorescence images were taken by inverted fluorescence microscope (Axio Observer Z1, ZEISS, Oberkochen, Germany).

## Conflict of Interest

The authors declare no conflict of interest.

## Author Contributions

The experiments were designed by L.K. and S.N. L.K. conducted photoelectrochemical measurements, fabricated and characterized the biointerfaces, and performed data analysis. O.K. and R.B. conducted patch‐clamp recordings and provided assistance with figure editing. H.N.K performed hippocampal neuron isolation, biocompatibility analysis and immunofluorescence imaging of hippocampal neurons. A.O. obtained X‐ray photoelectron spectroscopy data and interpreted it. S.A.V. interpreted electrochemical measurement results. M.H. supervised the neuron isolation, biocompatibility, immunofluorescence imaging, and provided data interpretation. The manuscript was written by L.K. and S.N. with contributions and confirmation from all authors.

## Supporting information

Supporting Information

## Data Availability

The data that support the findings of this study are available from the corresponding author upon reasonable request.
