# Supplementary material for: MnO2 Nanoflower Integrated Optoelectronic Biointerfaces for Photostimulation of Neurons
Source: Adv Sci (Weinh). 2023 Jun 29;10(25):2301854. doi: 10.1002/advs.202301854 (PMC10477844; doi:10.1002/advs.202301854)
Supplement: Supplementary file 1 — Supporting Information [file ADVS-10-2301854-s001.pdf]

ADVANCED SCIENCE
Open Access

Supporting Information

for *Adv. Sci.*, DOI 10.1002/advs.202301854

MnO_2 Nanoflower Integrated Optoelectronic Biointerfaces for Photostimulation of Neurons

*Lokman Kaya, Onuralp Karatum, Rıdvan Balamur, Hümeyra Nur Kaleli, Asım Önal, Sharadrao Anandrao Vanalakar, Murat Hasanreisoğlu and Sedat Nizamoglu**

Supporting Information

MnO_2 Nanoflower Integrated Optoelectronic Biointerfaces for Photostimulation of Neurons

Lokman Kaya[1], Onuralp Karatum[1], Rıdvan Balamur[1], Humeyra Nur Kaleli[2], Asım Önal[3], S. A. Vanalakar[4], Murat Hasanreisoğlu[2,5], Sedat Nizamoğlu[1,3,*]

[1] Department of Electrical and Electronics Engineering, Koc University, Istanbul, Turkey

[2] Research Center for Translational Medicine, Koc University, Istanbul, Turkey

[3] Department of Biomedical Science and Engineering, Koc University, Istanbul, Turkey

[4] Department of Physics, Karmaveer Hire College, Shivaji University, Gargoti, India

[5] Department of Ophthalmology, Medical School, Koc University, Istanbul, Turkey

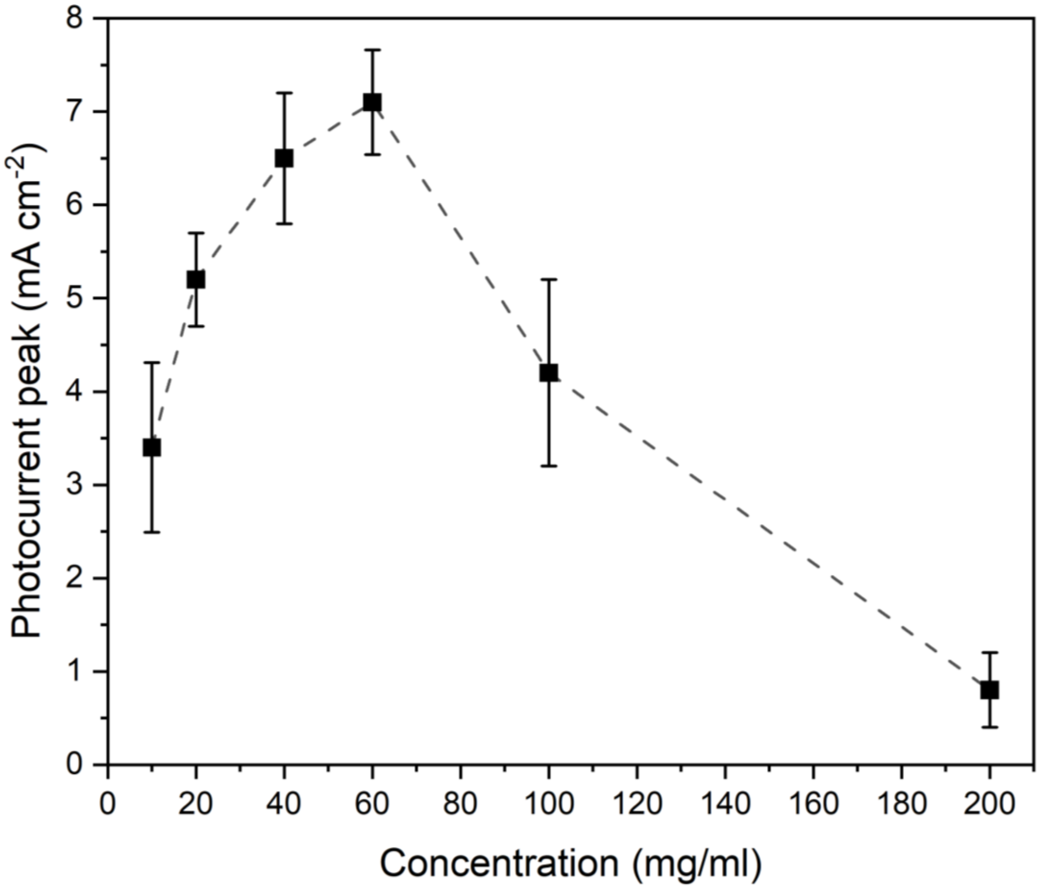

Figure S1. Optimization of peak photocurrent density by varying the spin-coated P3HT:PCBM concentration.

For optimization we varied the concentration of the coating solution of the photoactive layer while keeping the spin speed fixed at 1500 rpm (Figure S1). The increase of concentration directly affects the conversion of light to ionic currents and after the concentration of 60 mg ml^{-1} the photocurrent starts to drop possibly due to the limited electron and hole diffusion in thicker layers.

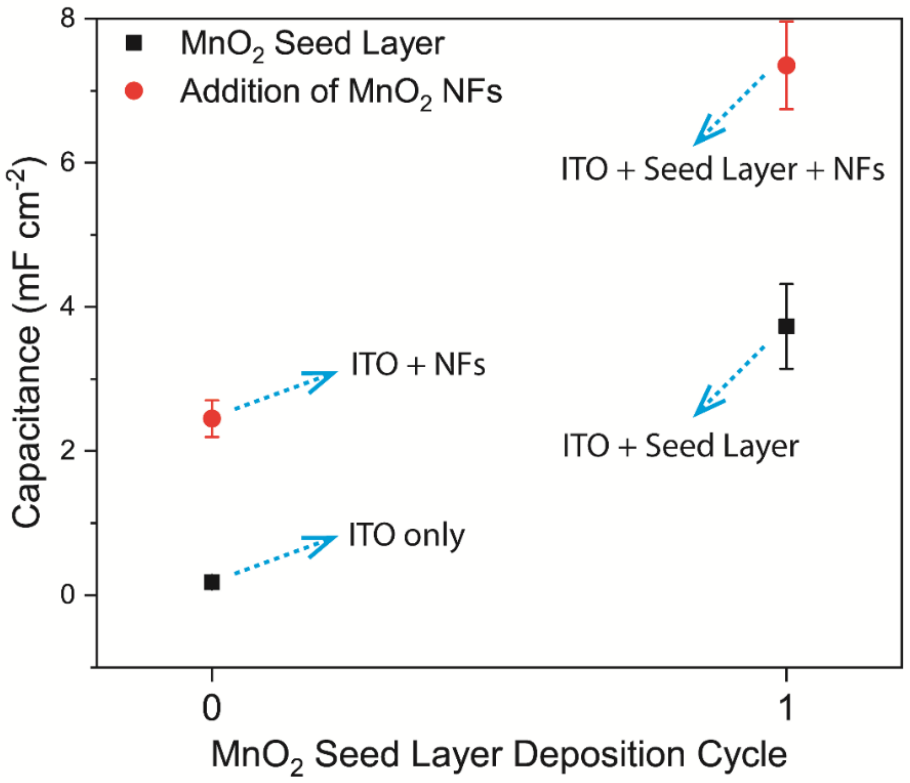

Figure S2. The effect of layers on the capacitance of various return electrodes.

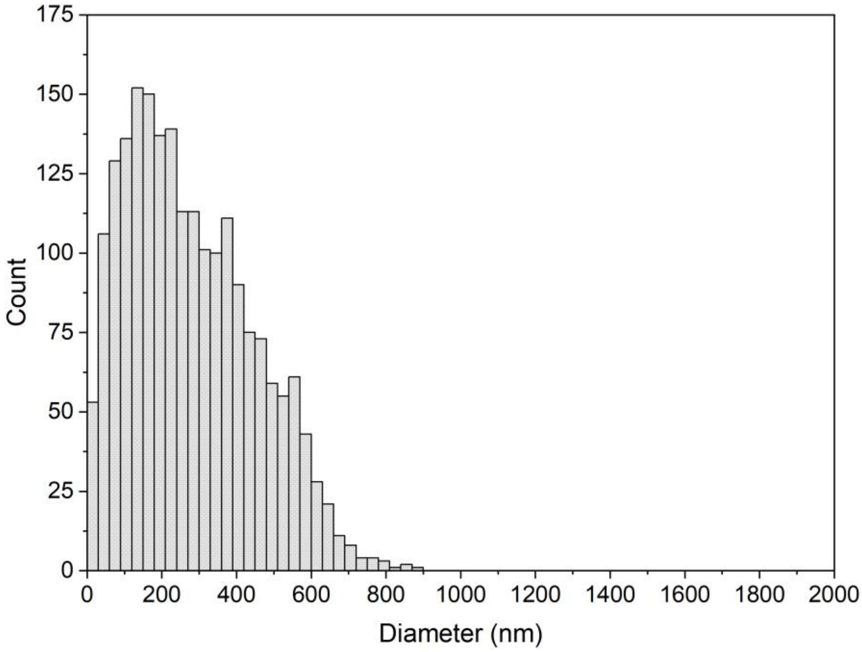

Figure S3. MnO_2 nanoflowers size distribution histograms obtained from SEM images (n>2000). According to Gaussian distribution, the mean is 279 nm, and the standard deviation is 169 nm.

Using our deposition technique, we observed nanoflowers of different sizes, and the average size is found to be 279 nm with a standard deviation of 169 nm (Figure S3).

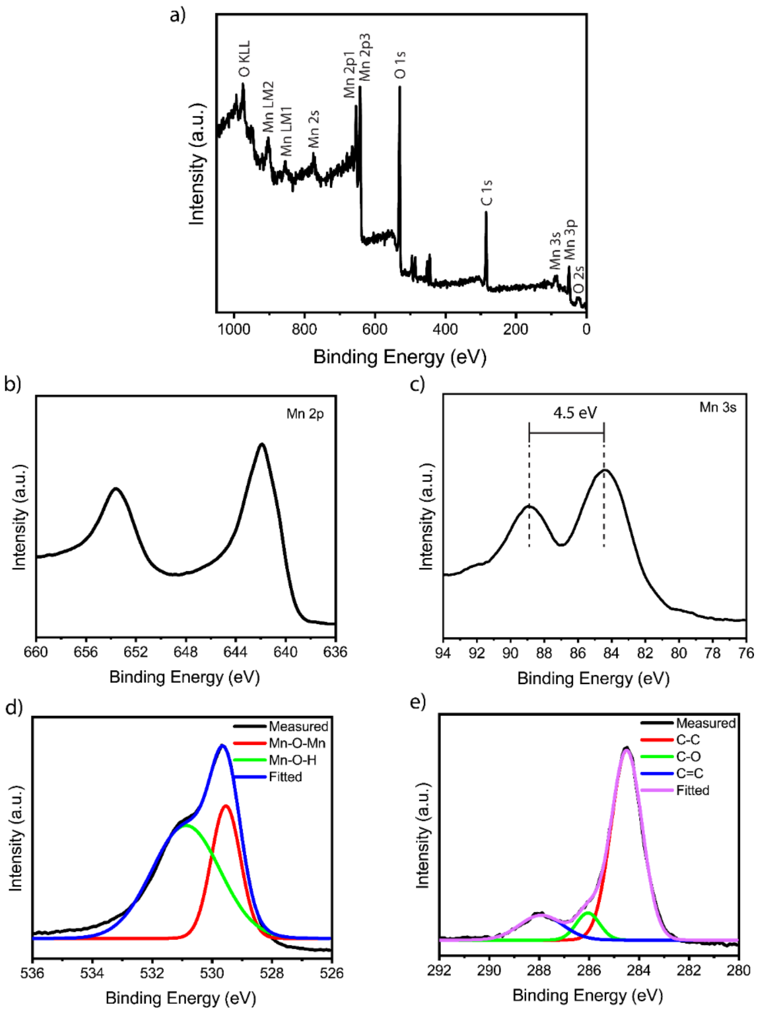

Figure S4. XPS spectra for MnO_2 nanoflowers a) survey spectrum and high-resolution spectra for b) Mn 2p, c) Mn 3s, d) O 1s, and e) C 1s.

According to the XPS survey spectrum (Figure S4a), there are 3 main elements, which are Mn, O, and C. The peaks at 495-486 eV, and 451.9-444.8 eV are attributed to the ITO substrate, which has Sn 3d and In 3d peaks. Figure S4b demonstrates the Mn 2p spectra of the manganese oxide flowers. Two signals are detected at around 641.9 and 653.6 eV due to the spin-orbit coupling [1], which can be ascribed to the binding energies of Mn $2p_{3/2}$ and Mn $2p_{1/2}$, respectively [2]. The energy difference between these signals is 11.7 eV, consistent with the previously reported data for Mn $2p_{3/2}$ and Mn $2p_{1/2}$ in MnO_2 [3]. Moreover, we performed XPS analysis on the Mn 3s region. In this analysis, we observed two distinct peaks at energy levels of 84.4 eV and 88.9 eV. The multiplet splitting, measured at 4.5 eV (Figure S4c), suggests the predominant presence of Mn (IV) oxidation states, in agreement with the previous literature [4] that indicates the existence of MnO_2. The high-resolution O 1s spectrum deconvoluted into two peaks, 529.6 and 531.1 eV, using the XPS best peak fitting with Gaussian modes (Figure S4d). These peaks can be attributed to Mn–O–Mn and Mn–O–H bonding according to the literature, respectively [5]. Also, the high-resolution C 1s spectrum of MnO_2 is shown in Figure S4.e C-C, C-O, and C=C peaks are deconvoluted at 284.5, 286.1, and 287.9 eV with the best fitting of Gaussian modes. During this analysis, all the peaks were adjusted based on the C 1s standard peak of 284.5 eV.

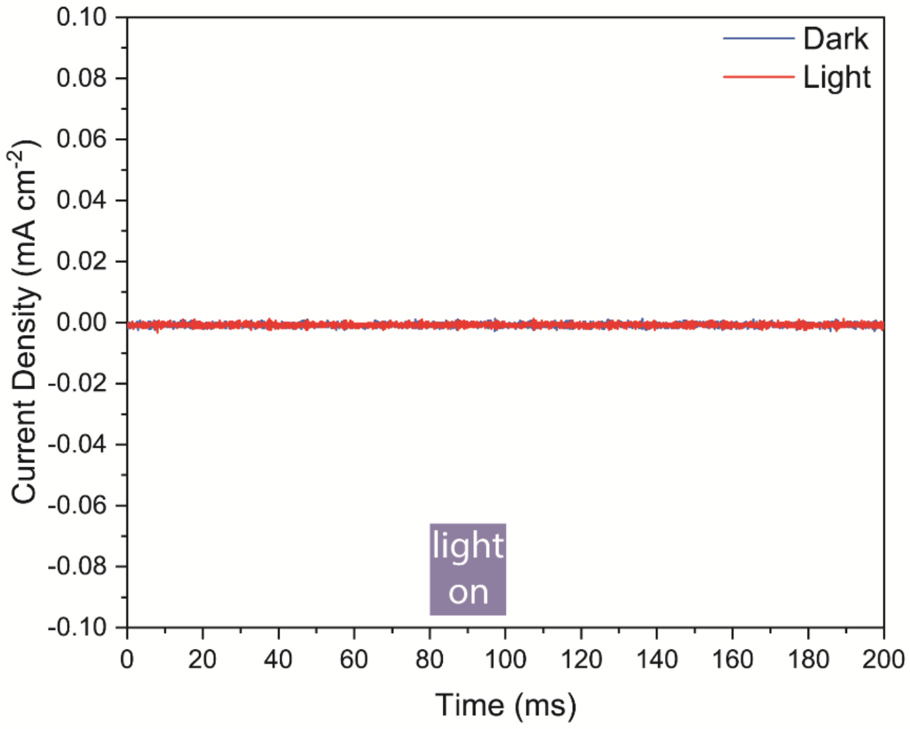

Figure S5. The current density of only MnO_2 return structure under the dark and light. The blue square represents the light pulse.

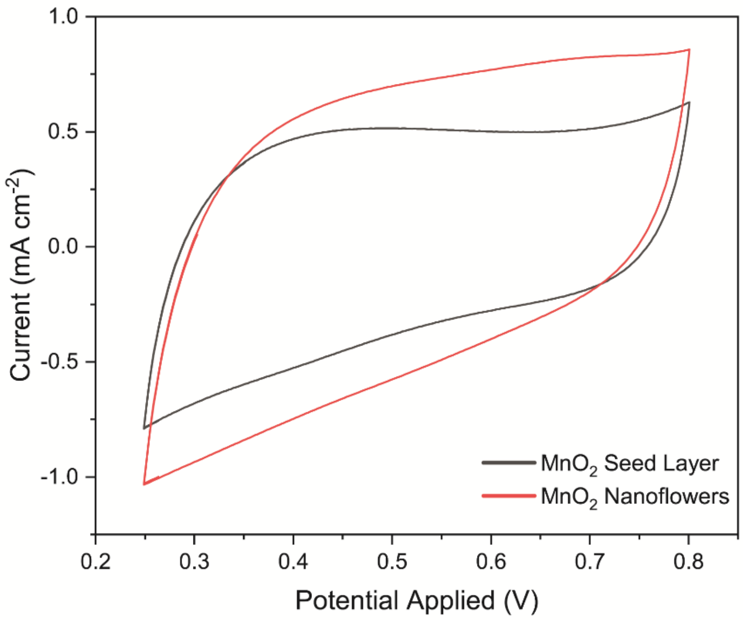

Figure S6. Cyclic voltammograms of MnO_2 seed layer and MnO_2 NFs on the seed layer (scan rate: 50 mV s^{-1}).

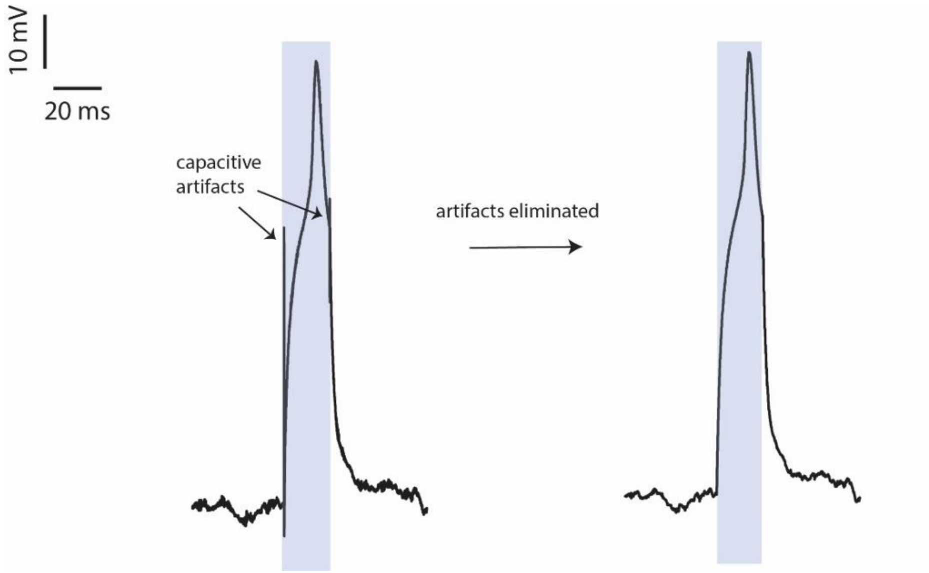

Figure S7. Current clamp recording trace showing the capacitive stimulation artifacts at the light onset and offset. Blue bars show the 20 ms 'light on' period. The artifacts are eliminated via downsampling and smoothing, which remains the characteristics of the action potential, such as threshold voltage, peak magnitude, and latency intact.

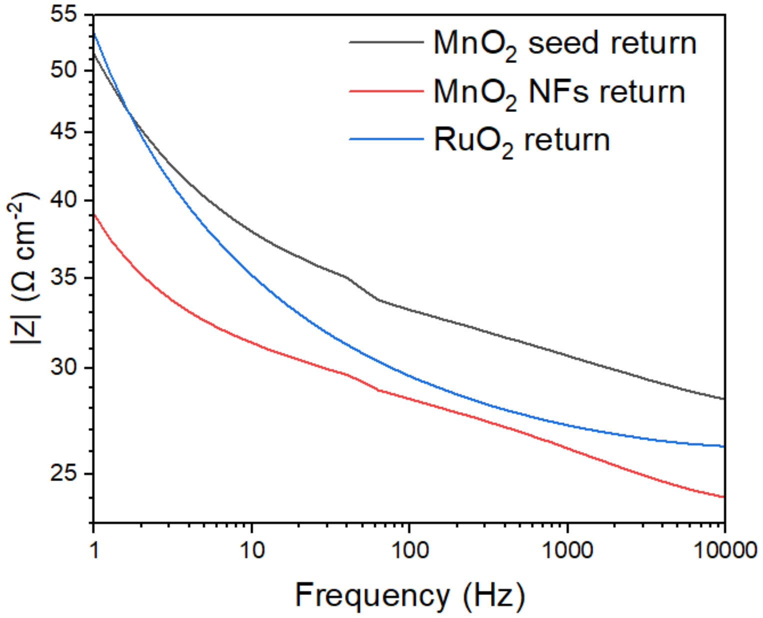

Figure S8. Impedance of MnO_2 seed, MnO_2 NFs and RuO_2 return in the frequency range of 1Hz - 10 kHz

Table S1. Photocurrent, charge density, excitation wavelength, light intensity and optical pulse duration in previous reports.

References	Photocurrent	Charge density	Wavelength	Light intensity or Light power	Optical pulse duration
**This study**	7.1 mA cm^{-2}	23.2 μC cm^{-2}	450 nm	0.99 mW mm^{-2}	20 ms
**[6]**	6 mA cm^{-2}	20 μC cm^{-2}	450 nm	0.87 mW mm^{-2}	10 ms
**[7]**	0.5 mA cm^{-2}	6 μC cm^{-2}	780 nm	1 mW mm^{-2}	20 ms
**[8]**	2 mA cm^{-2}	7.5 μC cm^{-2}	638 nm	8.5 mW mm^{-2}	5 ms
**[9]**	3 mA cm^{-2}	1 μC cm^{-2}	445 nm	1.5 mW mm^{-2}	50 ms
**[10]**	125 μA cm^{-2}	1.29 μC cm^{-2}	445 nm	0.57 mW mm^{-2}	10 ms
**[11]**	0.6 mA cm^{-2}	0.18 μC cm^{-2}	445 nm	1 mW mm^{-2}	20 ms
**[12]**	55 μA cm^{-2}	0.45 μC cm^{-2}	445 nm	0.57 mW mm^{-2}	10 ms
**[13]**	800 μA cm^{-2}	8 μC cm^{-2}	630 nm	0.33 mW mm^{-2}	10 ms
**[14]**	13 nA	8 pC	Warm white	180 mW	100 ms
**[15]**	4.5 nA	6.97 pC	445 nm	0.42 mW mm^{-2}	10 ms
**[16]**	135 μA cm^{-2}	-	565 nm	0.94 mW mm^{-2}	200 ms
**[17]**	200 pA	-	532 nm	15 mW mm^{-2}	20 ms
**[18]**	60 nA	-	530 nm	6 mW mm^{-2}	10 ms
**[19]**	400 μA	-	660 nm	0.6 mW mm^{-2}	5 ms
**[20]**	300 pA	-	450 nm	1.69 mW mm^{-2}	500 ms

Table S2. Deposition parameters of MnO_2 nanoflowers on different substrates.

		Electrochemical Deposition Parameters			Chemical Bath Deposition Parameters		
		Step Voltage (mV s^{-1})	Deposition Cycle	Annealing temperature (°C)	Bath Temperature (°C)	Bath Duration (Hour)	Annealing temperature (°C)
Substrate	**ITO**	**10**	**5**	**50**	**60**	**2**	**70**
	**FTO**	**12**	**6**	**80**	**70**	**2**	**90**
	**Gold**	**4**	**2**	**50**	**60**	**2**	**70**
	**Stainless steel**	**2**	**1**	**70**	**80**	**2**	**90**

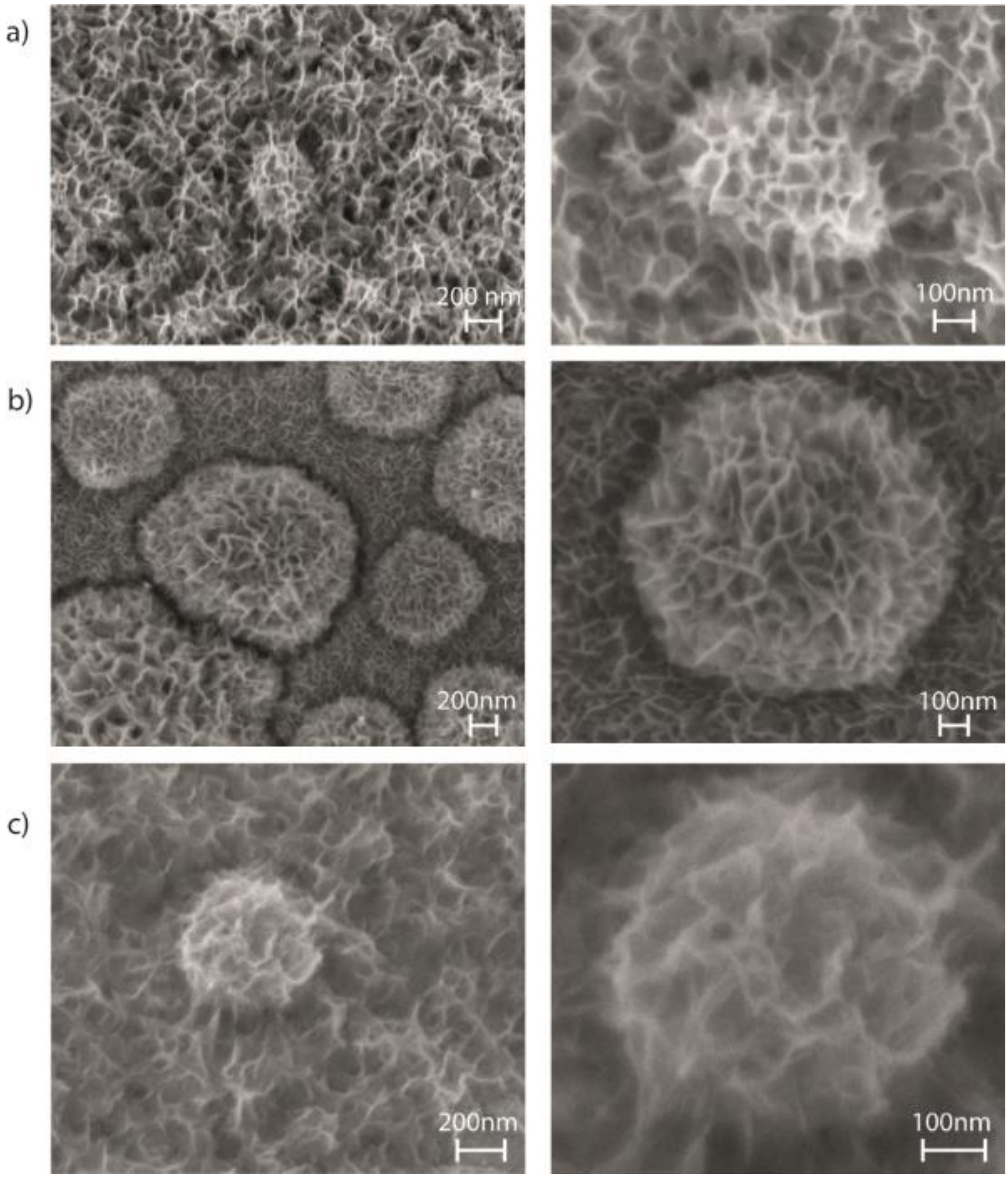

Figure S9. The SEM images of MnO_2 NFs coated on a) FTO, b) gold, and c) stainless steel substrates.

References

[1] I. I. Misnon, R. Abd Aziz, N. K. M. Zain, B. Vidhyadharan, S. G. Krishnan, R. Jose, *Materials Research Bulletin* **2014**, *57*, 221.

[2] a) M. Toupin, T. Brousse, D. Bélanger, *Chemistry of Materials* **2004**, *16* (16), 3184; b) W. Li, K. Xu, B. Li, J. Sun, F. Jiang, Z. Yu, R. Zou, Z. Chen, J. Hu, *ChemElectroChem* **2014**, *1* (6), 1003.

[3] Z. Li, Y. Mi, X. Liu, S. Liu, S. Yang, J. Wang, *Journal of Materials Chemistry* **2011**, *21* (38), 14706.

[4] E. Beyreuther, S. Grafström, L. M. Eng, C. Thiele, K. Dörr, *Physical Review B* **2006**, *73* (15), 155425.

[5] O. Sadak, W. Wang, J. Guan, A. K. Sundramoorthy, S. Gunasekaran, *ACS Applied Nano Materials* **2019**, *2* (7), 4386.

[6] O. Karatum, E. Yildiz, H. N. Kaleli, A. Sahin, B. Ulgut, S. Nizamoglu, *Advanced Functional Materials* **2022**, *32* (31), 2109365.

[7] O. Karatum, H. N. Kaleli, G. O. Eren, A. Sahin, S. Nizamoglu, *ACS Nano* **2022**, *16* (5), 8233, https://doi.org/10.1021/acsnano.2c01989.

[8] M. Silverå Ejneby, M. Jakešová, J. J. Ferrero, L. Migliaccio, I. Sahalianov, Z. Zhao, M. Berggren, D. Khodagholy, V. Đerek, J. N. Gelinas, *Nature Biomedical Engineering* **2022**, *6* (6), 741.

[9] M. Han, S. B. Srivastava, E. Yildiz, R. Melikov, S. Surme, I. B. Dogru-Yuksel, I. H. Kavakli, A. Sahin, S. Nizamoglu, *ACS applied materials & interfaces* **2020**, *12* (38), 42997.

[10] O. Karatum, M. M. Aria, G. O. Eren, E. Yildiz, R. Melikov, S. B. Srivastava, S. Surme, I. B. Dogru, H. Bahmani Jalali, B. Ulgut, *Frontiers in Neuroscience* **2021**, *15*, 652608.

[11] M. Han, H. Bahmani Jalali, E. Yildiz, M. H. Qureshi, A. Şahin, S. Nizamoglu, *Communications Materials* **2021**, *2* (1), 19.

[12] O. Karatum, G. O. Eren, R. Melikov, A. Onal, C. W. Ow-Yang, M. Sahin, S. Nizamoglu, *Scientific reports* **2021**, *11* (1), 2460.

[13] M. Silverå Ejneby, L. Migliaccio, M. Gicevičius, V. Đerek, M. Jakešová, F. Elinder, E. D. Głowacki, *Advanced Materials Technologies* **2020**, *5* (3), 1900860.

[14] O. S. Abdullaeva, F. Balzer, M. Schulz, J. Parisi, A. Lützen, K. Dedek, M. Schiek, *Advanced Functional Materials* **2019**, *29* (21), 1805177.

[15] R. Melikov, S. B. Srivastava, O. Karatum, I. B. Dogru-Yuksel, H. Bahmani Jalali, S. Sadeghi, U. M. Dikbas, B. Ulgut, I. H. Kavakli, A. E. Cetin, *ACS applied materials & interfaces* **2020**, *12* (32), 35940.

[16] L. Ferlauto, M. J. I. Airaghi Leccardi, N. A. L. Chenais, S. C. A. Gilliéron, P. Vagni, M. Bevilacqua, T. J. Wolfensberger, K. Sivula, D. Ghezzi, *Nature communications* **2018**, *9* (1), 992.

[17] D. Ghezzi, M. R. Antognazza, R. Maccarone, S. Bellani, E. Lanzarini, N. Martino, M. Mete, G. Pertile, S. Bisti, G. Lanzani, *Nature Photonics* **2013**, *7* (5), 400.

[18] Y. Jiang, X. Li, B. Liu, J. Yi, Y. Fang, F. Shi, X. Gao, E. Sudzilovsky, R. Parameswaran, K. Koehler, *Nature biomedical engineering* **2018**, *2* (7), 508.

[19] D. Rand, M. Jakešová, G. Lubin, I. Vėbraitė, M. David-Pur, V. Đerek, T. Cramer, N. S. Sariciftci, Y. Hanein, E. D. Głowacki, *Advanced Materials* **2018**, *30* (25), 1707292.

[20] H. Bahmani Jalali, O. Karatum, R. Melikov, U. M. Dikbas, S. Sadeghi, E. Yildiz, I. B. Dogru, G. Ozgun Eren, C. Ergun, A. Sahin, *Nano letters* **2019**, *19* (9), 5975.
